# Discovery of the genus *Nipponodipogon* Ishikawa in the Oriental region, with description of two new species from China (Hymenoptera, Pompilidae)

**DOI:** 10.3897/zookeys.692.12062

**Published:** 2017-08-21

**Authors:** Valery M. Loktionov, Arkady S. Lelej, Zai-fu Xu

**Affiliations:** 1 Federal Scientific Center of the East Asia Terrestrial Biodiversity, Far Eastern Branch of the Russian Academy of Sciences, Vladivostok-22 690022, Russia; 2 Department of Entomology, College of Agriculture, South China Agricultural University, Guangzhou 510640, China

**Keywords:** China, Deuterageniini, new species, *Nipponodipogon*, Oriental Region, Pepsinae

## Abstract

The genus *Nipponodipogon* Ishikawa, 1965 is newly recorded from China (Guangdong, Hainan, and Yunnan) and the Oriental Region. Two new species, *N. orientalis* Loktionov, Lelej & Xu, **sp. n.** (Guangdong, Hainan, Yunnan) and *N. shimizui* Loktionov, Lelej & Xu, **sp. n.** (Guangdong, Yunnan), are described and illustrated. The updated key to the species based on Shimizu et al. (2015) is given.

## Introduction

The family Pompilidae (spider wasps) is one of the largest families among the aculeate wasps in Hymenoptera. The family numbers around 5000 recent species in 125 genera and five subfamilies in the World (Aguiar et al. 2013, Waichert et al. 2015), 650 species in the Palaearctic (Lelej and Loktionov 2012a). The spider wasps are distributed worldwide, but mostly in the tropical regions (Pitts et al. 2006). The spider wasps are parasitoids that use spiders as host provisioning each cell with a single paralyzed spider on which they lay an egg (Iwata 1976). Some genera have evolved the mode of cleptoparasitism (Wasbauer 1995, Shimizu 2000, O’Neill 2001, Shimizu et al. 2012).

One of such cleptoparasitic genera is *Nipponodipogon* Ishikawa, 1965, a representative of brood parasitic wasps. Shimizu and Ishikawa (2002) pointed out the peculiar features in their antennal structure: the antenna is short, stout, and thickened toward middle of flagellum, and F2–F10 are somewhat flattened on the anteroventral side. Shimizu et al. (2012) confirmed the brood parasitism of *N. nagasei* and *N. iwatai* by using trap-nest technique. Based on several pieces of circumstantial evidence obtained from the contents of trap nests, they concluded that both species brood-parasitize species of *Deuteragenia* Šustera, 1912 (tribe Deuterageniini), and *N. iwatai* brood-parasitizes species of *Auplopus* Spinola, 1841 (tribe Auplopodini). They also discovered, that female of *N. nagasei* routinely lays up to five eggs on a single host spider, all of which develop into adult wasps without larval cannibalism; instead all spider wasps previously studied lay only one egg on a host spider (Shimizu et al. 2012).

*Nipponodipogon*, from the tribe Deuterageniini, subfamily Pepsinae, is distributed so far in the Eastern Palaearctic: in the Japanese Archipelago and the south of the Russian Far East. Ishikawa (1965) created this taxon as a subgenus of the genus *Dipogon* Fox, 1897, based on three species from Japan, Dipogon (Nipponodipogon) iwatai Ishikawa, 1965 (Honshu), D. (N.) nagasei Ishikawa, 1965 (Honshu and Kyushu) and D. (N.) mandibularis Ishikawa, 1965 (Honshu), the first of which is the type species. Later, Ishikawa (1968) described one species, D. (N.) hayachinensis Ishikawa from Japan, and Lelej (1986) described two species: D. (N.) rossicus Lelej and D. (N.) kurilensis Lelej from the Russian Far East. In the phylogenetic analysis of the tribe Deuterageniini (Lelej and Loktionov 2012b), *Nipponodipogon*, as well as, other subgenera of the genus *Dipogon* were proposed as separated genera. Shimizu et al. (2015) revised the genus *Nipponodipogon*, and described *N. sudai* Shimizu from Japan. Before this study, the genus included seven species that have been known from Japan and the Russian Far East (Loktionov and Lelej 2014, Shimizu et al. 2015).

In this paper we describe two new species of *Nipponodipogon* from China and enlarge the distribution of the genus to include China and the Oriental Region.

## Materials and methods

During the study of hymenopteran collection in South China Agricultural University, we examined more than 2300 specimens of Chinese spider wasps collected during last two decades from Jilin, Inner Mongolia, Ningxia, Gansu, Shaanxi, Henan, Zhejiang, Hebei, Fujian, Hunan, Guangdong, Hainan, Guangxi, Yunnan, Sichuan, and Guizhou. Of them only 14 specimens belonging to the genus *Nipponodipogon* were collected in 2006, 2010, and 2011 years in the Oriental part of China (Guangdong, Hainan and Yunnan) by yellow pan traps and sweeping nets. The following acronyms are used for the collections where type specimens are deposited:

**IBSS** Federal Scientific Center of the East Asia Terrestrial Biodiversity, Far Eastern Branch of the Russian Academy of Sciences (former Institute of Biology and Soil Science), Vladivostok, Russia (curator Prof. Arkady Lelej).

**SCAU** Hymenopteran Collection of South China Agricultural University, Guangzhou, China (curator Prof. Zai-fu Xu).

To study male genitalic characters, genitalia were extracted after being previously softened. The muscles were removed in a sodium hydroxide solution (NaOH 10%). The genitalia were later placed in water to neutralize the NaOH and stored in micro vials filled with glycerin. Male genitalia were studied under a stereomicroscope in a depression slide.

Photographs of imagos and genitalia were taken with stereomicroscope SteREO Discovery.V12 and stacked using CombineZM software (Hadley 2008). The final illustrations were post-processed for contrast and brightness using Adobe® Photoshop® software.

The terminology for morphology is mostly based on the glossary provided by the Hymenoptera Anatomy Consortium (2013) and Shimizu et al. (2015). The terminology of wing venation and cells follows Day (1988). The following abbreviations are used for morphological terms:

**F1**, **F2**, **F3** etc., the first, second, third flagellomeres, etc.;

**MID** the middle interocular distance;

**OOD** the distance between posterior ocellus and compound eye which is measured from above;

**POD** the postocellar distance which is measured from above;

**S1**, **S2**, **S3** etc., the first, second, third metasomal sterna, etc.;

**SMC2** the second submarginal cell of fore wing;

**SMC3** the third submarginal cell of fore wing;

**T1**, **T2**, **T3** etc., the first, second, third metasomal terga etc.;

**UID** the upper interocular distance.

## Systematics

### Genus Nipponodipogon

Taxon classificationAnimaliaHymenopteraPompilidae

Ishikawa, 1965

Dipogon (Nipponodipogon) Ishikawa, 1965: 89. Type species: Dipogon (Nipponodipogon) iwataiIshikawa 1965, ♀ (Japan: Honshu), by original designation.
Nipponodipogon : Lelej and Loktionov 2012a: 413; 2012b: 11; Loktionov and Lelej 2014: 153; Shimizu et al. 2015: 498.

#### Diagnosis.

*Female*. Maxillary cardo with a few thin, pale bristles, the apex of these not extending beyond the maxillary lacinia. Antenna short, stout, and thickened toward middle of flagellum (fusiform); F1 less than 3× its width. Supra-antennal area of frons produced anteriorly into a frontal ledge overhanging the antennal radicle. Apical margin of labrum not or slightly emarginated medially. Metapleuron strongly convex above level of lateral face of pronotum and metapleuron (dorsal view). Metapostnotum narrow and practically linear, deeply sunken between the metanotum and propodeum. Crossvein *cu-a* of hind wing short and almost straight, forming obtuse angle with vein *1A*. *Male*. Antenna slightly thickened medially, usually with F3–F11 triangularly produced beneath (except for *N. orientalis* Loktionov, Lelej & Xu, sp. n. and *N. shimizui* Loktionov, Lelej & Xu, sp. n.); F1 1.3–2.0× its width. Mandible with one subapical inner tooth. Body punctate. Exposed portion of hypopygium stick-like, compressed laterally; subbasal portion strongly widened (Figs 21, 42, 48).

#### Species included.

Nine species. *Nipponodipogon hayachinensis* (Ishikawa, 1968), ♀ (Japan: Honshu); *N. iwatai* (Ishikawa, 1965), ♀ & ♂ (Japan: Hokkaido and Honshu); *N. kurilensis* (Lelej, 1986), ♀ (Russia: Kuril Islands); *N. mandibularis* (Ishikawa, 1965), ♀ (Japan: Honshu); *N. nagasei* (Ishikawa, 1965), ♀ & ♂ (Japan: Hokkaido, Honshu and Kyushu); *N. rossicus* (Lelej, 1986), ♀ & ♂ (Russia: Primorskij Terr.); *N. sudai* Shimizu *in* Shimizu, Lelej & Loktionov, 2015, ♀ & ♂ (Japan: Hokkaido and Honshu) (Shimizu et al. 2015 and Shimizu and Terayama 2016); *N. orientalis* Loktionov, Lelej & Xu, sp. n., ♀ & ♂ (China: Guangdong, Hainan and Yunnan); *N. shimizui* Loktionov, Lelej & Xu, sp. n., ♀ & ♂ (China: Guangdong and Yunnan).

#### Distribution.

Palaearctic Region (Russia: Primorskij Terr., Kuril Islands; Japan: Hokkaido, Honshu, Kyushu) and Oriental Region (new record) (China: Guangdong, Hainan, Yunnan).

#### Biology.

The representatives of the genus *Nipponodipogon* are brood parasitic wasps. *Nipponodipogon nagasei* and *N. iwatai* brood-parasitize species of *Deuteragenia* Šustera, 1912 (tribe Deuterageniini), and *N. iwatai* brood-parasitizes species of *Auplopus* Spinola, 1841 (tribe Auplopodini). Female of *N. nagasei* routinely lays up to five eggs on a single host spider, all of which develop into adult wasps without larval cannibalism, instead all spider wasps previously studied lay only one egg on a host spider (Shimizu et al. 2012).

### Nipponodipogon orientalis

Taxon classificationAnimaliaHymenopteraPompilidae

Loktionov, Lelej & Xu sp. n.

http://zoobank.org/006909A8-2FEC-4B94-95AF-766C7B128E5F

Figs 1 , 2–5 , 6–10 , 11 , 12–17 , 18–22

#### Material examined.

**Holotype**. CHINA: ♀, Guangdong, Nankunshan, 4–6.VI.2011, Zai-fu Xu, No. 2016001247 (SCAU). **Paratypes**. CHINA: 2 ♀, with the same data as holotype, No. 2016001227 and 2016001217 (SCAU); 1 ♀, with the same data as holotype, No. 2016001255 (SCAU); 1 ♀, Hainan, Diaoluoshan, 12–13.VII.2010, Hua-yan Chen, No. 2016000370 (SCAU); 1 ♂, Yunnan, Gaoligongshan, 20–21.VII.2006, Zai-fu Xu, No. 2016000480 (IBSS); 1 ♂, Yunnan, Gaoligongshan, 20–21.VII.2006, Zai-fu Xu, No. 2016000479 (SCAU).

#### Diagnosis.

**Figure 1.**
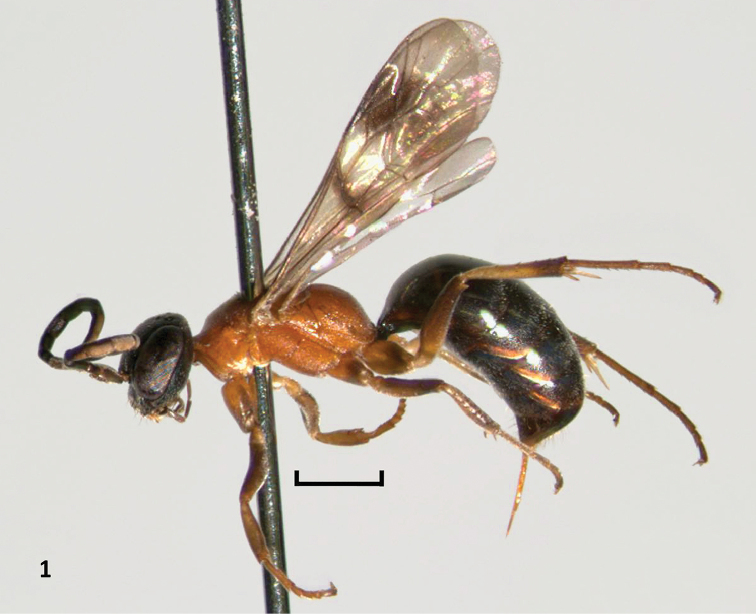
*Nipponodipogon orientalis* Loktionov, Lelej & Xu, sp. n., female, holotype, habitus, lateral view. Scale bar 1 mm.

*Female*. Mesosoma completely yellow orange (Figs 1, 7). Posterolateral portion of propodeum with strong transverse rugae (Figs 5–7). T1 with long petiole basally (Fig. 6). Outer apicoventral corner of the metafemur produced triangularly (Fig. 8). *Male*. T1 distinctly petiolate basally (Fig. 15). F3–F11 not produced triangularly beneath, not forming serrated profile. Propodeum matt, with weak dense transverse striae posterolaterally (Fig. 15). Subbasal portion of hypopygium with round sublateral carina (Fig. 21, arrow).

#### Description.

***Female***. Body length 6.1–8.0 mm; fore wing length 4.7–6.2 mm. Head and metasoma black; sometimes clypeus along anterior margin brownish; antenna black, except flagellomeres 3–10 muddy yellow ventrally and sometimes scape and pedicel yellowish-brown ventrally; mandible brownish subapically. Mesosoma completely yellow orange (Figs 1, 7). Legs brown with abundant yellowish-brown (Fig. 1) to completely muddy yellowish. Fore wing weakly infuscate or sometimes more or less yellowish, with distinct two fuscous bands (Fig. 9). Hind wing weakly infuscate (Fig. 10).

**Figures 2–5.**
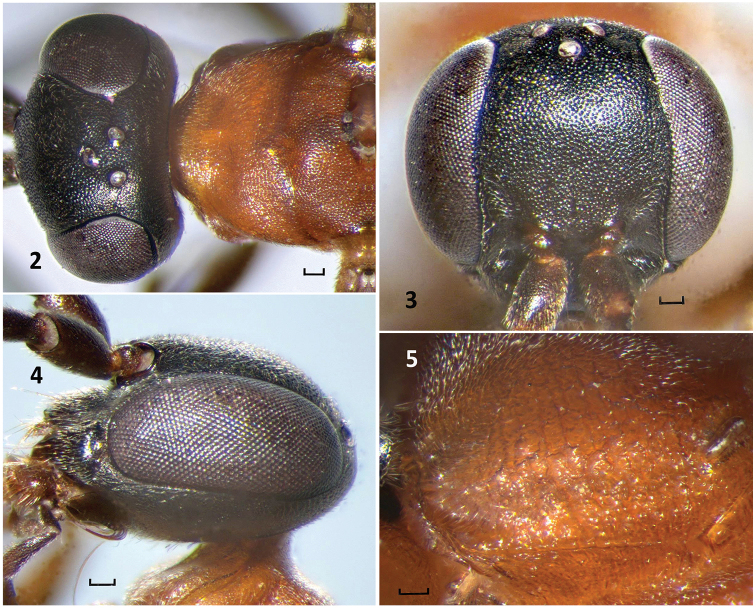
*Nipponodipogon orientalis* Loktionov, Lelej & Xu, sp. n., female, paratype. **2** Head and pronotum, dorsal view **3** Head, frontal view **4** Head, lateral view **5** Propodeum, dorsolateral view. Scale bars 0.1 mm.

**Figures 6–10.**
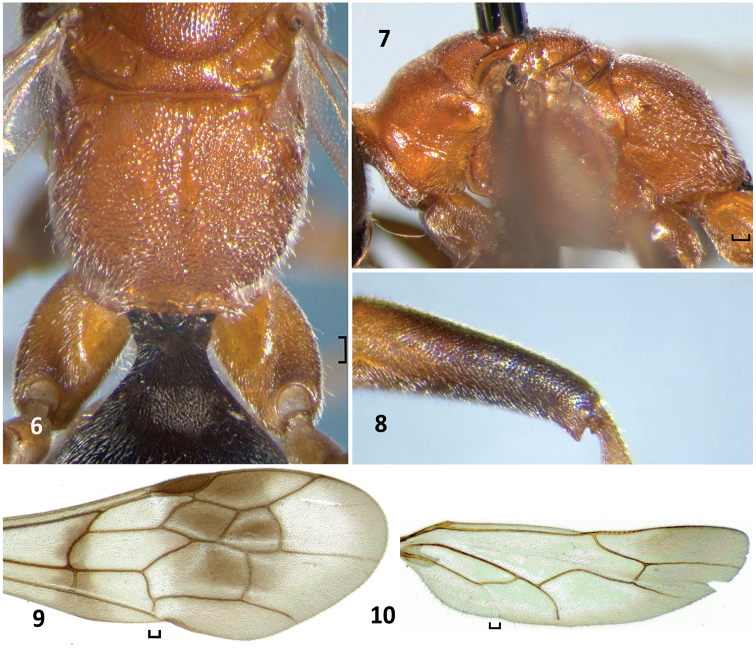
*Nipponodipogon orientalis* Loktionov, Lelej, Xu, sp. n., female, paratype. **6** Mesoscutellum, metanotum, metapostnotum, propodeum and T1, dorsal view **7** Mesosoma, lateral view **8** Metafemur, outer lateral view **9** Fore wing **10** Hind wing. Scale bars 0.1 mm.

*Head and mesosoma* matt. Frons, vertex, and mesosoma, except propodeum, finely and densely punctate. Pronotum anteriorly, laterally and collar finely striate and punctate. Mesopleuron with denser punctures. Upper mesopleuron and metapleuron finely and densely striate. Lateral side of metanotum with several regular oblique striae. Propodeum strongly and densely punctate with fine transverse rugae posteriorly and much stronger rugae posterolaterally (Figs 5–7). Metasoma somewhat polished. T1–T5 with fine punctures; T6 and S6 less polished than other segments, with scattered setiferous pores located on all exposed portion; S1–S5 with somewhat larger punctures than on T1–T5. S1 with several longitudinal rugae baso-medially. Transverse groove on S2 gently arcuate.

*Body* with gray pubescence mostly short, but longer on clypeus, mesopleuron, propodeum posterolaterally and coxae. Body without setae except the following: upper frons sometimes with one long erect setae and a few shorter ones; clypeus with a few long suberect setae anteriorly; coxae and T1 basally with scattered short erect setae; S2–S5 with scattered longer erect setae posteriorly; T6 and S6 with denser long erect pale setae.

Width of *head* in frontal view 1.1–1.2× its height. Vertex weakly convex between eye tops (Fig. 3). Upper frons gently convex (Fig. 4). Frons without median line, but sometimes with indistinct elongate concavity medially. Supra-antennal area of frons produced anteriorly into a frontal ledge overhanging the antennal radicle (Fig. 4). Inner orbits weakly convergent above and subparallel below (Fig. 3). Half of MID 1.3–1.4× eye width. Ocelli large, slightly raised; ocellar triangle acute-angled (Fig. 2); POD/OOD=0.8–1.1. Posterior margin of vertex roundly concave (dorsal view) (Fig. 2). Clypeus convex medially with distinct concavity basolaterally; anterolateral corner broadly rounded; anterior margin almost straight or weakly emarginate medially; width 2.7–2.9× its length. Apical margin of labrum broadly rounded. Mandible with subapical tooth. Maxillary cardo with two tufts of thin, light brown bristles. Malar space short. Gena strongly narrowing posteriorly (Fig. 2, dorsal view; Fig. 4, lateral view). Antenna short, stout, and thickened toward middle of flagellum; F1–F3 distinctly widening toward apex; apex of apical flagellomere pointed; F1 length 0.95–1.0× F2 length; F1 length 2.2–2.4× its width and 0.50–0.65× UID.

*Pronotum* with anterior declivity flattened, not distinctly differentiated from dorsum; dorsum in dorsal view slightly narrowing anteriorly; shoulder gently rounded; juncture between dorsal and lateral faces narrowly and roundly raised; posterior margin weakly and arcuately emarginate medially. Mesoscutum slightly sloped anteriorly; posterolateral margin not reflexed; parapsidal sulcus finely impressed. Discs of mesoscutellum and metanotum barely raised above level of mesoscutum and propodeum (Fig. 7). Metapostnotum narrow and practically linear, deeply sunken between metanotum and propodeum (Fig. 6). Propodeum evenly convex with flattened posterior declivity not well differentiated from dorsum (Fig. 7).

*Fore wing* (Fig. 9) with SMC2 receiving crossvein *1m-cu* at almost middle; SMC3 1.1–1.2× longer than SMC2 on vein *M*, and 0.6–0.7× longer than SMC2 on vein *Rs*; receiving crossvein *2m-cu* at almost middle; crossvein *2rs-m* almost straight or sometimes barely curved; crossvein *3rs-m* distinctly curved; crossvein *cu-a* barely postfurcal. Hind wing (Fig. 10). Outer apicoventral corner of metafemur produced triangularly (Fig. 8). Claws symmetrical with large subapical inner tooth. T1 distinctly petiolate (Fig. 6). S6 with a longitudinal median rounded carina posteriorly.

**Figure 11.**
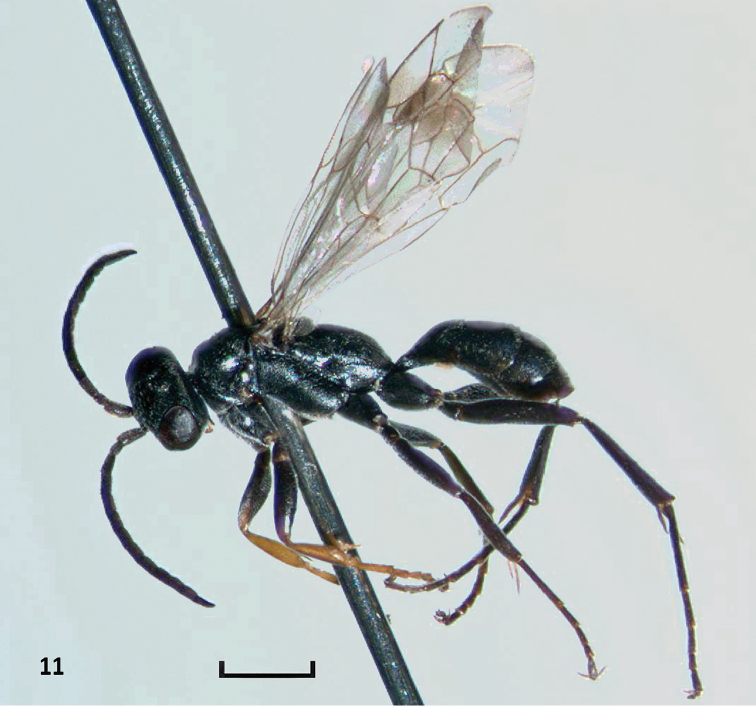
*Nipponodipogon orientalis* Loktionov, Lelej & Xu, sp. n., male, paratype, habitus, lateral view. Scale bar 1 mm.

***Male***. Body length 3.7–4.6 mm; fore wing length 3.5–4.1 mm. Body black; antenna black with scape brown or black ventrally and flagellum weakly brown ventrally; mandible brown subapically; pro- tibia and tarsi brown; spurs of pro- and mesotibia brown, spurs of metatibia dark brown (Fig. 11). Fore wing weakly infuscate, with weak subapical fuscous band (Fig. 16). Hind wing weakly infuscate (Fig. 17).

*Body* mostly punctate and somewhat polished. Frons, discs of pronotum, mesoscutum, mesoscutellum, and metanotum finely and densely punctate. Pronotum laterally polished and indistinctly punctate. Mesopleuron with coarser punctures than frons. Upper mesopleuron striate. Lateral side of metanotum with several regular oblique striae. Metapleuron finely punctate. Propodeum more or less matt, finely and densely punctate with weak dense transverse striae posterolaterally. Metasomal segments finely punctate. S1 with several longitudinal rugae basally. Transverse groove on S2 weak, gently arcuate, not connected medially. S6 with scattered setiferous pores (Fig. 18). Body with gray pubescence mostly short, but longer on lower face, clypeus, propleuron, propodeum posteriorly and mesepisternum. Body without setae except the following: upper frons with one long erect setae; T7 and S6 with long erect brown setae.

**Figures 12–17.**
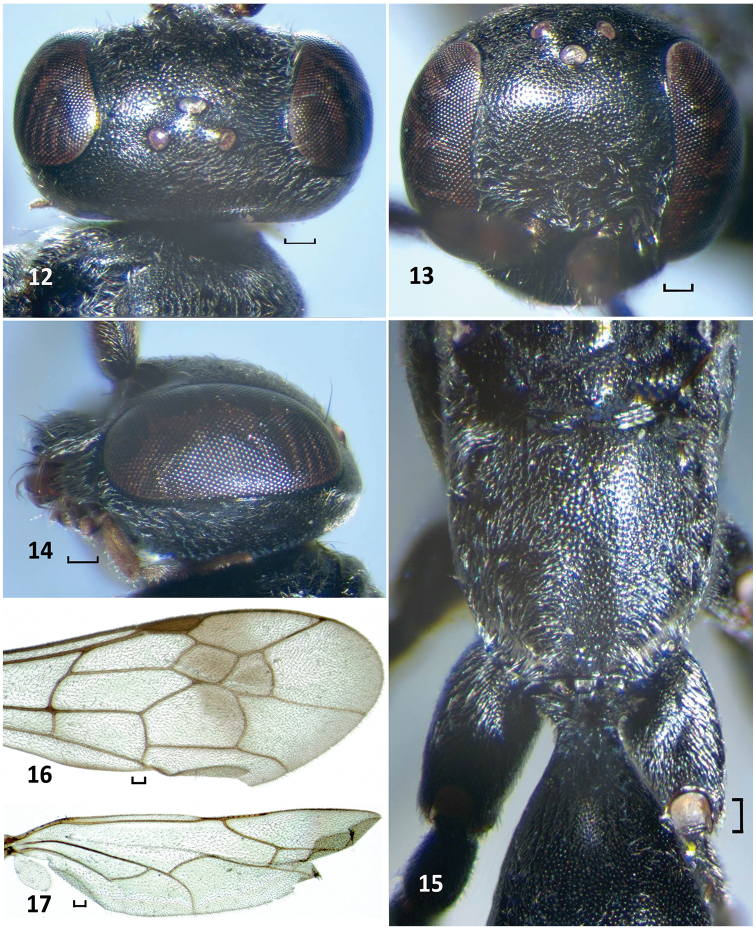
*Nipponodipogon orientalis* Loktionov, Lelej & Xu, sp. n., male, paratype. **12** Head, dorsal view **13** Head, frontal view **14** Head, lateral view **15** Mesoscutellum, metanotum, metapostnotum, propodeum and T1, dorsal view **16** Fore wing **17** Hind wing. Scale bars 0.1 mm.

Width of *head* in frontal view 1.1× its height. Vertex moderately convex between eye tops (Fig. 13). Upper frons gently convex (Fig. 14). Frons without median line, with indistinct elongate concavity medially. Supra-antennal area of frons produced anteriorly into weak frontal ledge overhanging the antennal radicle (Fig. 14). Inner orbits subparallel above and barely convergent below (Fig. 13). Half of MID 1.4–1.6× eye width. Ocelli large, noticeably raised; ocellar triangle right-angled (Fig. 12); POD/OOD=0.75–0.85. Posterior margin of vertex straight (dorsal view) (Fig. 12). Clypeus weakly convex medially; anterolateral corner broadly rounded; anterior margin barely broadly rounded, almost straight medially. Mandible with subapical tooth. Malar space short. Gena narrowing posteriorly (Fig. 12, dorsal view; Fig. 14, lateral view). Antenna shortened; flagellum filiform; flagellomeres indistinctly convex ventrally, not forming triangle projection; apex of apical flagellomere pointed; F1 length 0.9–1.0× F2 length; F1 length 1.85–1.90× its width and 0.30–0.36× UID.

*Pronotum* with anterior declivity weakly concave, more differentiated from dorsum than in female; dorsum in dorsal view narrowing anteriorly; shoulder gently rounded; juncture between dorsal and lateral faces roundly raised; posterior margin weakly and arcuately emarginate. Mesoscutum slightly sloped anteriorly; parapsidal sulcus finely impressed. Discs of mesoscutellum and metanotum somewhat more strongly raised above level of mesoscutum and propodeum than in female. Metapostnotum (Fig. 15) longer and not deeply sunken between metanotum and propodeum, as in female; somewhat narrowing in middle; metapostnotum length 0.15–0.25× metanotum length medially. Propodeum evenly convex with posterior declivity not differentiated from dorsum; posterior surface evenly convex.

**Figures 18–22.**
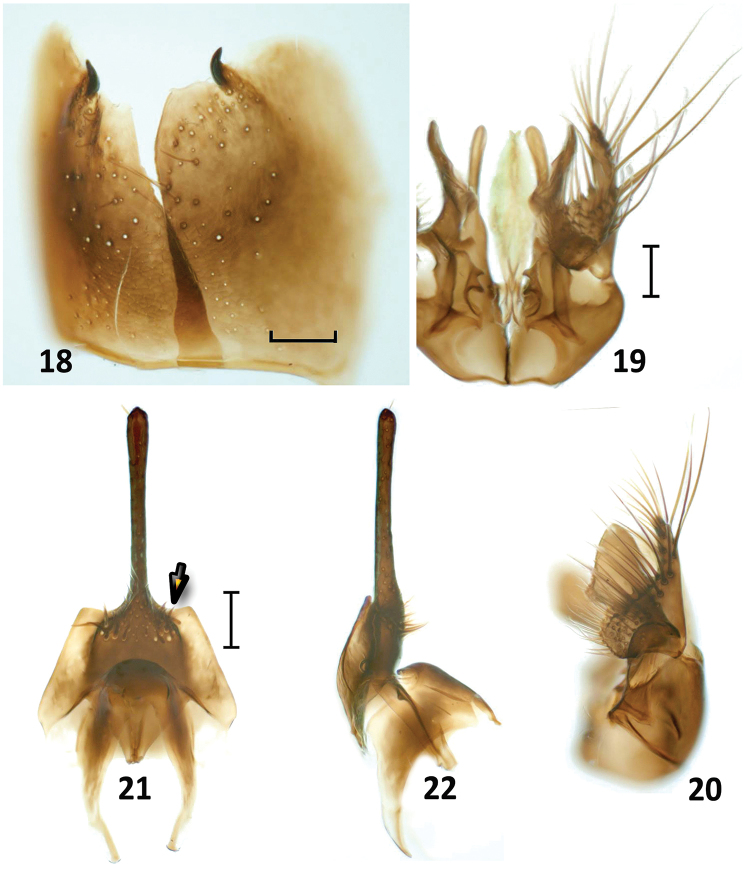
*Nipponodipogon orientalis* Loktionov, Lelej & Xu, sp. n., male, paratype. **18** S6, ventral view **19** Genitalia, ventral view **20** Genitalia, lateral view **21** Hypopygium and S7, ventral view **22** Hypopygium and S7, lateral view. Scale bars 0.1 mm.

*Fore wing* (Fig. 16), hind wing (Fig. 17). Claws symmetrical with large subapical inner tooth. T1 distinctly petiolate; petiole long (Fig. 15). S6 deeply and arcuately emarginate posteriorly; lateral hook small, curved and pointed to apex (Fig. 18). Exposed portion of hypopygium stick form, compressed laterally, narrow (both in lateral and ventral views), weakly widened apically; subbasal portion extended laterally, with short erected stout spines (Figs 21, 22). Paramere broadly widened basally and narrowing toward apex (lateral view), with long bristles, longer bristle as long as paramere; volsella broad apically (lateral view) (Figs 19, 20).

#### Remarks.

**Figure 23.**
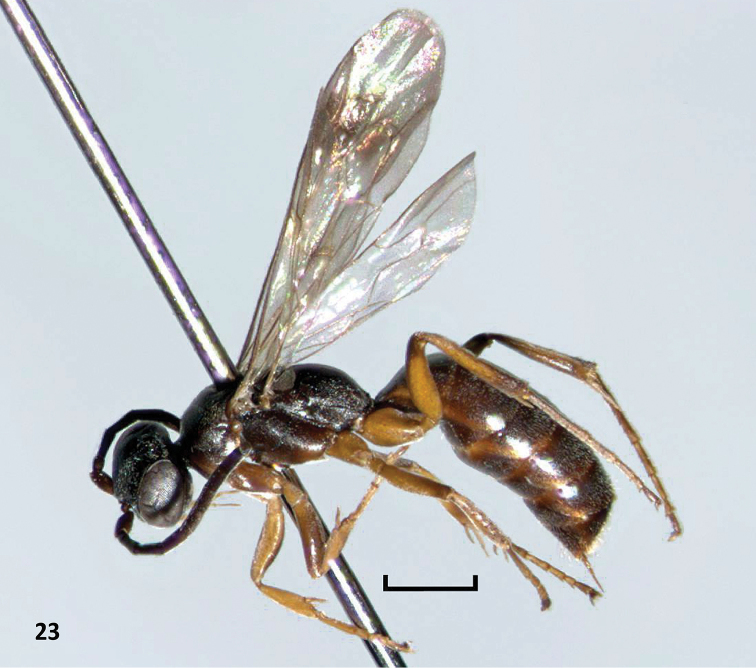
*Nipponodipogon shimizui* Loktionov, Lelej & Xu, sp. n., female, holotype, habitus, lateral view. Scale bars 1 mm.

The female of new species is similar to those of *Nipponodipogon kurilensis*, *N. sudai*, and *N. shimizui* sp. n. by having outer apicoventral corner of metafemur produced triangularly (Fig. 8) and T1 petiolate basally (Fig. 6), but can be separated from all of them in having mesosoma completely yellow orange (Figs 1, 7) (*vs* completely or mostly black (Figs 23, 29)) and posterolateral portion of propodeum with strong transverse rugae (Figs 5–7) (*vs* with fine transverse striae or/and punctures (Figs 27–29, 44)). Female of *N. orientalis* sp. n. differs from that of *N. kurilensis* in having T1 with long petiole (Fig. 6) (*vs* short one in *N. kurilensis* (Shimizu et al. 2015: fig. 3D)); and from that of *N. shimizui* sp. n. in having crossvein *3rs-m* distinctly curved (Fig. 9) and T6 somewhat polished, not shagreened, with distinct scattered setiferous pores (*vs* crossvein *3rs-m* almost straight and T6 matt, shagreened, without distinct setiferous pores in *N. shimizui* sp. n. (Figs 31, 39)).

Male of new species is closely related to that of *N. shimizui* sp. n. by some morphological characters including shape of hypopygium and genitalia, but easily distinguishes in propodeum with fine transverse striae posterolaterally (Fig. 15) (*vs* propodeum without any striae in *N. shimizui* sp. n. (Fig. 38)); exposed portion of hypopygium narrow in lateral view (Fig. 22) (*vs* noticeably wider in *N. shimizui* sp. n. (Fig. 43)); subbasal portion of hypopygium in ventral view with round sublateral carina (Fig. 21, arrow) (*vs* with angulate sublateral carina in *N. shimizui* sp. n. (Fig. 42, arrow)); S6 with setiferous pores posteromedially (Fig. 18) (*vs* without setiferous pores posteromedially in *N. shimizui* sp. n. (Fig. 39)). Male of new species is also similar to that of *N. sudai* in having petiole on T1 basally (Fig. 15), but can be easily differentiated by having F3–F11 not producing triangularly beneath, not forming serrated profile (*vs* F3–F11 produced triangularly beneath, forming serrated profile in *N. sudai*); lateral hook on S6 small, claw-like, curved and pointed to apex (Fig. 18) (*vs* lateral hook on S6 large, strongly compressed laterally and thin, subtriangular in profile in *N. sudai* (Fig. 46)); and exposed portion of hypopygium without long erect setae (Figs 21, 22) (*vs* with long erect setae in *N. sudai* (Fig. 48)).

#### Sex association.

In spite of the fact that females and males were collected in different locations (two males from Yunnan and five females from Guangdong and Hainan) and have different coloration (mesosoma completely yellow orange in female *vs* completely black in male), we consider that they are opposite sexes of same species. Male of new species has propodeum with fine transverse striae posterolaterally that correlates with strong transverse rugae on propodeum posteriorly, especially in posterolateral portion in female (*vs* male without any striae, female with fine transverse striae in *Nipponodipogon shimizui* sp. n.). Such coloration differences in female and male of new species are not exception and occur in widely distributed Palaearctic species *Arachnotheutes rufithorax* (Costa, 1881) (Loktionov and Lelej 2017: figs 87, 88).

#### Etymology.

The name of the new species refers to the first record of the genus in the Oriental Region.

#### Distribution.

China (Guangdong, Hainan, Yunnan).

### Nipponodipogon shimizui

Taxon classificationAnimaliaHymenopteraPompilidae

Loktionov, Lelej & Xu sp. n.

http://zoobank.org/C4D684DE-E576-49AD-8300-B403CE1E5F78

Figs 23 , 24–27 , 28–32 , 33 , 34–38 , 39–43

#### Material examined.

**Holotype**. CHINA: ♀, Guangdong, Nanling, 8–17.VIII.2010, Hua-yan Chen, yellow pan traps, No. 2016001839 (SCAU). **Paratypes**. CHINA: 3 ♀, with the same data as holotype, No. 2016001836, 2016001840 and 2016001842 (SCAU); 1 ♀, with the same data as holotype, No. 2016001837 (IBSS); 1 ♀, Guangdong, Nanling, 5–7.VI.2010, Hua-yan Chen, No. 2016000023 (SCAU); 1 ♂, Yunnan, Lushui, 19.VII.2006, Zai-fu Xu, No. 2016000326 (SCAU).

#### Diagnosis.

*Female*. Outer apicoventral corner of metafemur produced triangularly (Fig. 30). T1 with distinct petiole basally (Fig. 28). Crossvein *2rs-m* almost straight or sometimes barely curved; crossvein *3rs-m* straight or almost straight (Fig. 31). Mesoscutum raised along midline (Fig. 29). Head and mesosoma matt; metasoma somewhat polished. *Male*. T1 distinctly petiolate basally (Fig. 38). F3–F11 not produced triangularly beneath, not forming serrated profile. Propodeum polished, without any striae (Fig. 38). Subbasal portion of hypopygium with angulate sublateral carina (Fig. 42, arrow).

#### Description.

***Female***. *Body* length 5.2–6.4 mm; fore wing length 4.3–5.1 mm. Head, mesosoma and metasoma black; sometimes clypeus along anterior margin dark brown; antenna black, except F3–F10 muddy yellow ventrally and scape yellowish-brown ventrally; mandible brownish subapically. Legs yellowish-brown or brown with procoxa laterally, profemur externally, meso- and metafemur, tibiae apically and tarsi somewhat darker (Fig. 23). Fore wing weakly infuscate, with weak subbasal and preapical fuscous bands (Fig. 31). Hind wing weakly infuscate (Fig. 32).

*Head and mesosoma* matt. Frons, vertex and mesosoma, except propodeum, finely and densely punctate. Pronotum laterally and finely striate and punctate. Mesopleuron with denser and coarser punctures then on disc of pronotum. Upper mesopleuron rugose. Metapleuron finely and densely striate. Lateral side of metanotum with several regular oblique striae. Propodeum strongly and densely punctate with fine transverse rugae posteriorly. Metasoma somewhat polished, except T6 and S6 matt. T1–T5 with fine punctures; T6 finely shagreened, without distinct setiferous pores; S6 less shagreened, than T6, with scattered setiferous pores located posteriorly and postero-laterally; S1–S5 with somewhat larger punctures than on T1–T5. S1 with several longitudinal rugae medially. Transverse groove on S2 gently arcuate.

*Body* with gray pubescence mostly short, but longer on propodeum posterolaterally. Body without setae except the following: upper frons sometimes with one long erect setae; clypeus with a few long suberect setae anteriorly; S2–S5 with scattered long or short erect setae posteriorly; T6 and S6 with denser long erect pale setae.

**Figures 24–27.**
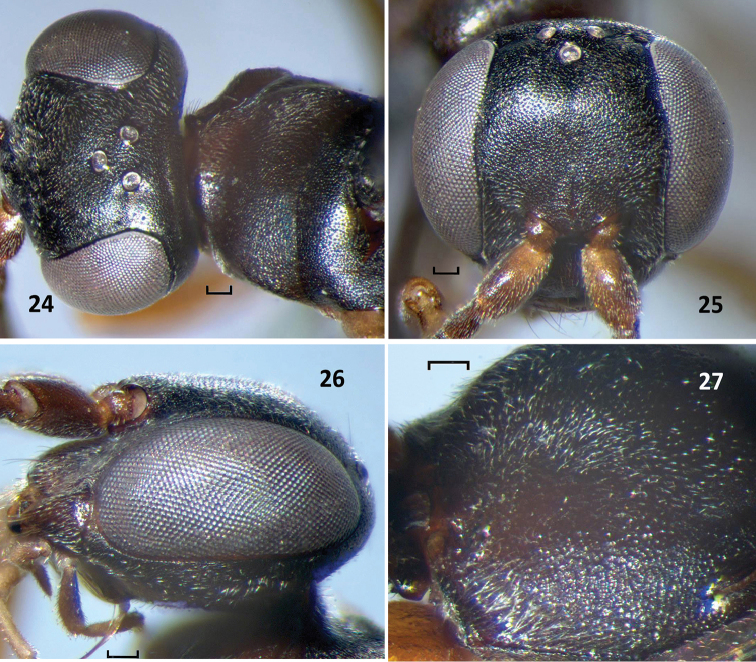
*Nipponodipogon shimizui* Loktionov, Lelej & Xu, sp. n., female, paratype. **24** Head and pronotum, dorsal view **25** Head, frontal view **26** Head, lateral view **27** Propodeum, dorsolateral view. Scale bars 0.1 mm.

Width of *head* in frontal view 1.1–1.2× its height. Vertex weakly convex between eye tops (Fig. 25). Upper frons gently convex (Fig. 26). Frons with indistinct median line and fine elongate concavity medially. Supra-antennal area of frons produced anteriorly into a frontal ledge overhanging the antennal radicle (Fig. 26). Inner orbits weakly convergent above and subparallel below (Fig. 25). Half of MID 1.3–1.6× eye width. Ocelli large, slightly raised; ocellar triangle barely acute-angled (Fig. 24); POD/OOD=0.6–0.8. Posterior margin of vertex roundly concave (dorsal view) (Fig. 24). Clypeus convex medially with distinct concavity basolaterally; anterolateral corner broadly rounded; anterior margin almost straight or weakly emarginate medially; width 2.7× its length. Apical margin of labrum broadly rounded. Mandible with large subapical tooth and indistinct basal tooth. Maxillary cardines with two tufts of thin, light brown bristles. Malar space short. Gena narrowing posteriorly (Fig. 24, dorsal view; Fig. 26, lateral view). Antenna short, stout, and thickened toward middle of flagellum; F1–F4 distinctly widening toward apex; apex of apical flagellomere pointed; F1 length 0.90–0.95× F2 length; F1 length 2.2–2.6× its width and 0.5× UID.

**Figures 28–32.**
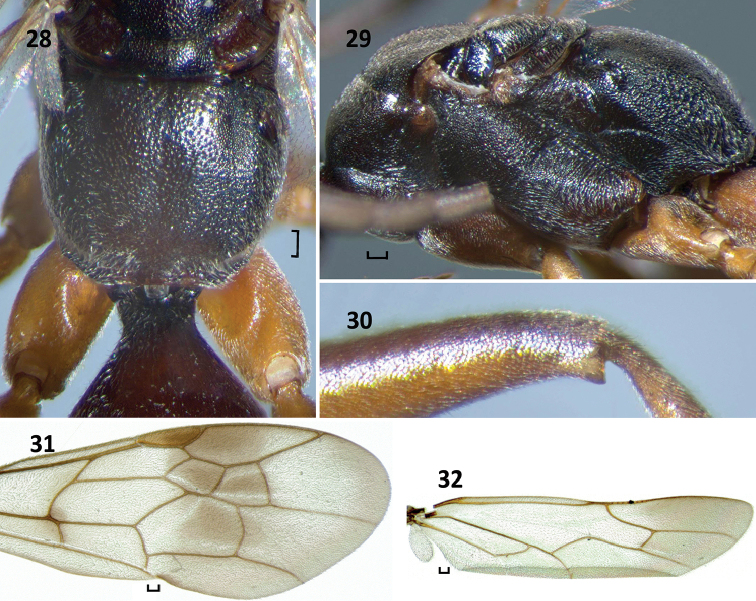
*Nipponodipogon shimizui* Loktionov, Lelej & Xu, sp. n., female, paratype. **28** Mesoscutellum, metanotum, metapostnotum, propodeum and T1, dorsal view **29** Mesosoma, lateral view **30** Metafemur, outer lateral view **31** Fore wing **32** Hind wing. Scale bars 0.1 mm.

*Pronotum* with anterior declivity flattened, not distinctly differentiated from dorsum; dorsum in dorsal view slightly narrowing anteriorly; shoulder gently rounded; juncture between dorsal and lateral faces narrowly and roundly raised; posterior margin weakly and arcuately emarginate medially (Fig. 24). Mesoscutum slightly sloped anteriorly; disc along median line slightly convex; posterolateral margin not reflexed; parapsidal sulcus finely impressed. Discs of mesoscutellum and metanotum barely raised above level of mesoscutum and propodeum (Fig. 29). Metapostnotum narrow and practically linear, deeply sunken between metanotum and propodeum (Fig. 28). Propodeum evenly convex with flattened posterior declivity not well differentiated from dorsum (Fig. 29).

*Fore wing* (Fig. 31) with SMC2 receiving crossvein *1m-cu* at almost middle; SMC3 1.2–1.5× longer than SMC2 on vein *M*, and 0.8–1.1× longer than SMC2 on vein *Rs*; receiving crossvein *2m-cu* at almost middle; crossvein *2rs-m* almost straight or sometimes barely curved; crossvein *3rs-m* straight, sometimes barely curved; crossvein *cu-a* barely postfurcal. Hind wing (Fig. 32). Outer apicoventral corner of metafemur produced triangularly (Fig. 30). Claws symmetrical with large subapical inner tooth. T1 distinctly petiolate (Fig. 28). S6 with a longitudinal median rounded carina posteriorly.

**Figure 33.**
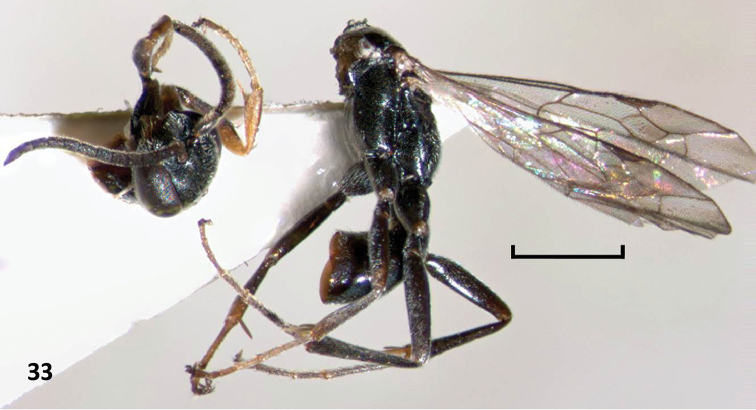
*Nipponodipogon shimizui* Loktionov, Lelej & Xu, sp. n., male, paratype, habitus, lateral view. Scale bar 1 mm.

***Male***. *Body* length 3.8 mm; fore wing length 3.4 mm. Body black; antenna black with scape brown ventro-apically and flagellum indistinctly brownish ventrally; mandible brown subapically; protibia and protarsi brown; spurs of tibia brown (Fig. 33). Fore wing weakly infuscate, with darker apical portion, fuscous band indistinct (Fig. 37). Hind wing weakly infuscate. Body mostly punctate and somewhat polished. Frons, discs of pronotum, mesoscutum, mesoscutellum, metanotum finely and densely punctate. Pronotum laterally polished and indistinctly punctate. Mesopleuron with coarser punctures than frons. Upper mesopleuron without striate. Lateral side of metanotum with several regular oblique striae. Metapleuron indistinctly punctate. Propodeum basolaterally polished with fine punctures larger than on frons, without any striae. Metasomal segments finely punctate. S1 with several longitudinal rugae basally. Transverse groove on S2 weak. S6 lacking setiferous pores posteromedially (Fig. 39). Body with gray pubescence mostly short, but longer on lower face, clypeus, and propodeum posteriorly. Body without setae except upper frons with one long erect setae and clypeus with a few long suberect setae anteriorly.

**Figures 34–38.**
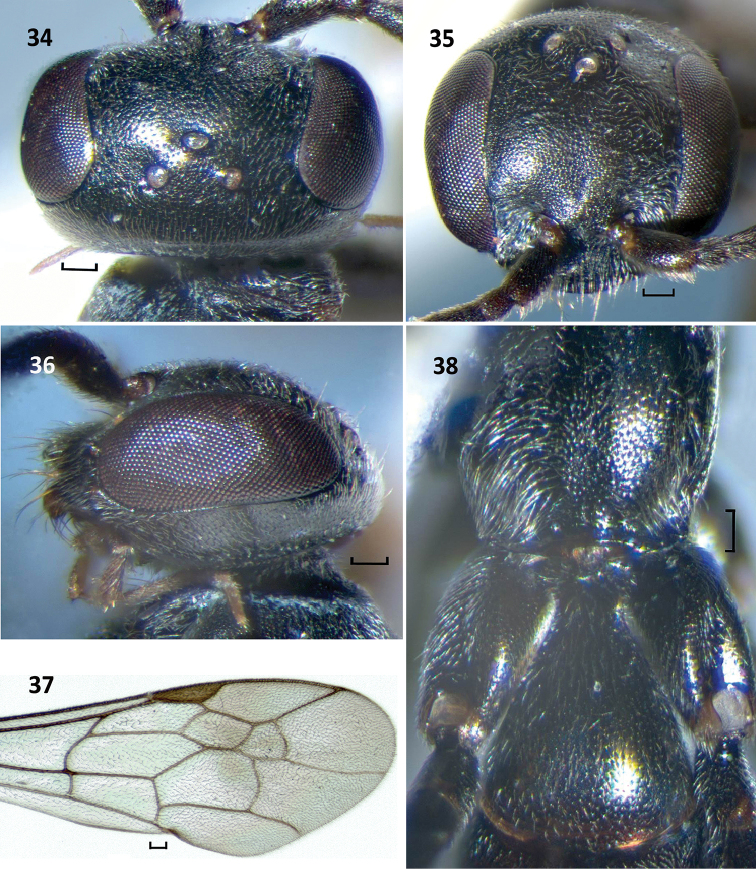
*Nipponodipogon shimizui* Loktionov, Lelej & Xu, sp. n., male, paratype. **34** Head, dorsal view **35** Head, frontal view **36** Head, lateral view **37** Fore wing **38** Propodeum and T1, dorsal view. Scale bars 0.1 mm.

Width of *head* in frontal view 1.1× its height. Vertex moderately convex between eye tops (Fig. 35). Upper frons gently convex (Fig. 36). Frons without median line, with indistinct elongate concavity medially. Supra-antennal area of frons produced anteriorly into weak frontal ledge overhanging antennal radicle (Fig. 36). Inner orbits subparallel above and barely convergent below (Fig. 35). Half of MID 1.6× eye width. Ocelli large, noticeably raised; ocellar triangle right-angled (Fig. 34); POD/OOD=0.9. Posterior margin of vertex straight (dorsal view) (Fig. 34). Clypeus weakly convex medially; anterolateral corner rounded; anterior margin broadly rounded. Mandible with subapical tooth. Malar space short. Gena weakly narrowing posteriorly (Fig. 34, dorsal view; Fig. 36, lateral view). Antenna shortened; flagellum filiform; flagellomeres indistinctly convex ventrally, not forming triangle projection; apex of apical flagellomere pointed; F1 length 1.0× F2 length; F1 length 1.8× its width and 0.3× UID.

*Pronotum* with anterior declivity weakly concave, more differentiated from dorsum than in female; dorsum in dorsal view narrowing anteriorly; shoulder gently rounded; juncture between dorsal and lateral faces roundly raised; posterior margin arcuately emarginate. Parapsidal sulcus finely impressed. Discs of mesoscutellum and metanotum somewhat stronger raised above level of mesoscutum and propodeum than in female. Metapostnotum longer and not deeply sunken between metanotum and propodeum, as in female; somewhat narrowing in middle; metapostnotum length 0.25× metanotum length medially. Propodeum evenly convex with posterior declivity not differentiated from dorsum; posterior surface evenly convex.

**Figures 39–43.**
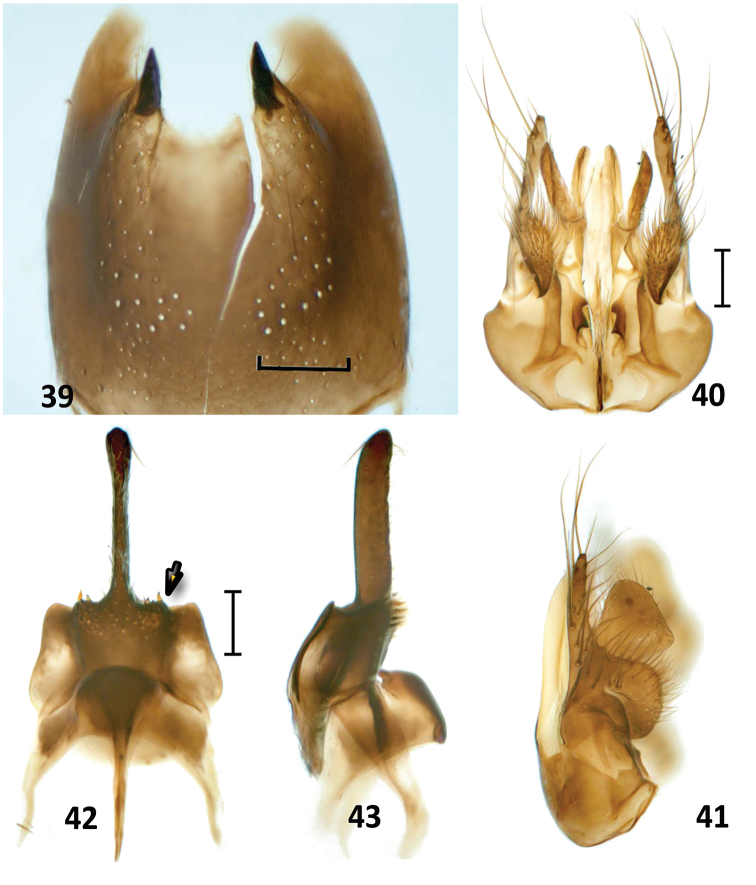
*Nipponodipogon shimizui* Loktionov, Lelej & Xu, sp. n., male, paratype. **39** S6, ventral view **40** Genitalia, ventral view **41** Genitalia, lateral view **42** Hypopygium and S7, ventral view **43** Hypopygium and S7, lateral view. Scale bars 0.1 mm.

*Fore wing* (Fig. 37). Claws symmetrical with small subapical inner tooth. T1 distinctly petiolate (Fig. 38). S6 deeply and arcuately emarginate posteriorly; lateral hook barely curved and pointed to apex (Fig. 39). Exposed portion of hypopygium stick form, compressed laterally, narrow and widened apically (ventral view); subbasal portion extended laterally, with short stout erect spines on two angulate sublateral carinae (Figs 42, 43). Paramere broadly widened basally and strongly narrowing toward apex (lateral view), with long bristles, longer bristle 0.7× longer than paramere; volsella broad apically (lateral view) (Figs 40, 41).

#### Remarks.

**Figures 44–48.**
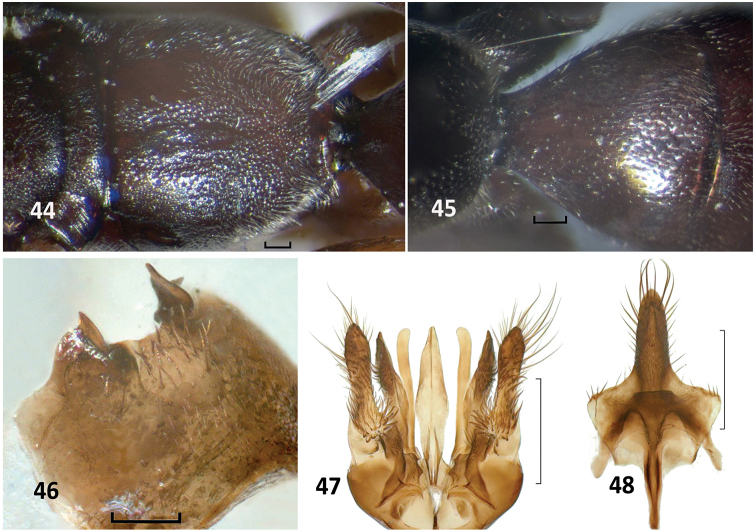
*Nipponodipogon sudai* Shimizu, paratype. **44** Mesoscutellum, metanotum, metapostnotum, propodeum and base of T1, dorsal view **45** T1, dorsal view **46** S6, ventral view **47** Genitalia, ventral view **48** Hypopygium and S7, ventral view **44** Female **45–48** Male. Scale bars 0.1 mm for **44–46**; 0.25 mm for **47, 48**.

The female of new species is similar to those of *Nipponodipogon kurilensis*, *N. sudai* and *N. orientalis* sp. n. by having outer apicoventral corner of metafemur produced triangularly (Fig. 30) and T1 petiolate basally (Fig. 28), but can be distinguished from them by following characters: posterolateral portion of propodeum with fine transverse striae and punctures (Figs 27–29) (*vs* with strong transverse rugae in *N. orientalis* sp. n. (Figs 5, 7)); mesosoma completely black (Figs 23, 29) (*vs* completely yellow orange in *N. orientalis* sp. n. (Figs 1, 7)); T6 matt and shagreened, without distinct setiferous pores (*vs* somewhat polished, not shagreened, with distinct scattered setiferous pores in *N. orientalis* sp. n.); vertex between eye tops slightly convex (Fig. 25) (*vs* distinctly convex in *N. kurilensis* (Shimizu et al. 2015: fig. 3A)); petiole of T1 long (Fig. 28) (*vs* very short in *N. kurilensis* (Shimizu et al. 2015: fig. 3D)); head and mesosoma matt, metasoma somewhat polished (*vs* head and mesosoma somewhat polished, metasoma distinctly polished in *N. kurilensis*); mesoscutum raised along midline (Fig. 29) (*vs* not raised in *N. sudai* (Shimizu et al. 2015: fig. 8D)); crossvein *3rs-m* almost straight (Fig. 31) (*vs* gently or moderately curved in *N. sudai* (Shimizu et al. 2015: fig. 9J)); propodeum anteromedially punctate (Fig. 28) (*vs* not punctate in *N. sudai* (Fig. 44)).

Male of new species is closely related to that of *N. orientalis* sp. n. by having some morphological characters including shape of hypopygium and genitalia, but can be easily distinguished in having propodeum without any striae posterolaterally (Fig. 38) (*vs* with fine transverse striae posterolaterally in *N. orientalis* sp. n. (Fig. 15)); exposed portion of hypopygium noticeably wider in lateral view (Fig. 43) (*vs* narrow in *N. orientalis* sp. n. (Fig. 22); subbasal portion of hypopygium in ventral view with angulate sublateral carina (Fig. 42, arrow) (*vs* with round sublateral carina in *N. orientalis* sp. n. (Fig. 21, arrow)); and S6 without setiferous pores posteromedially (Fig. 39) (*vs* with setiferous pores in *N. orientalis* sp. n. (Fig. 18)). Male of new species is also similar to that of *N. sudai* in having petiole in T1 basally (Fig. 38), but can be separated in having F3–F11 not producing triangularly beneath, not forming serrated profile (*vs* F3–F11 produced triangularly beneath, forming serrated profile in *N. sudai*); lateral hook on S6 claw-like, weakly curved and pointed to apex (Fig. 39) (*vs* lateral hook on S6 strongly compressed laterally and thin, subtriangular in profile in *N. sudai* (Fig. 46)); and exposed portion of hypopygium without long erect setae (Figs 42, 43) (*vs* with long erect setae in *N. sudai* (Fig. 48)).

#### Sex association.

In spite of females and males were collected in different locations (one male in Yunnan and six females in Guangdong), we consider that they are opposite sexes of the same species. Male S6 of new species lacks setiferous pores posteromedially (Fig. 39), which correlates with female S6 of similar condition medially (*vs* with scattered setiferous pores in male and female of *Nipponodipogon orientalis* sp. n.).

#### Etymology.

It is a pleasure to name this species after the well-known taxonomist Dr. Akira Shimizu (Tokyo Metropolitan University, Japan).

#### Distribution.

China (Guangdong, Yunnan).

### The updated key of *Nipponodipogon* species

(based on Shimizu et al. 2015)

**Females**

**Table:** 

1	Outer apicoventral corner of metafemur produced triangularly (Figs 8, 30). T1 petiolate basally (Figs 6, 28, 44)	**2**
–	Outer apicoventral corner of metafemur rounded (Shimizu et al. 2015: fig. 2D). T1 not petiolate basally (Shimizu et al. 2015: fig. 6E)	**5**
2	Posterolateral portion of propodeum with strong transverse rugae (Figs 5, 7). Mesosoma completely yellow orange (Figs 1, 7)	***N. orientalis* Loktionov, Lelej & Xu, sp. n.**
–	Posterolateral portion of propodeum with fine transverse striae or punctures (Figs 27–29, 44). Mesosoma completely black (Figs 23, 29), sometimes posterior margin of pronotum and posterolateral margin of metapostnotum brownish	**3**
3	Vertex between eye tops strongly convex (Shimizu et al. 2015: fig. 3A). Petiole of T1 very short (Shimizu et al. 2015: fig. 3D). Head and mesosoma somewhat polished; metasoma distinctly polished. Ocelli forming right-angle triangle (Shimizu et al. 2015: fig. 3B)	***N. kurilensis* (Lelej)**
–	Vertex between eye tops slightly convex (Fig. 25). Petiole of T1 long (Fig. 28). Head and mesosoma matt; metasoma not distinctly polished. Ocelli usually forming acute-angle triangle (Fig. 24)	**4**
4	Mesoscutum not raised along midline (Shimizu et al. 2015: fig. 7D). Crossvein *3rs-m* gently or moderately curved (Shimizu et al. 2015: fig. 9J). Disc of propodeum without punctures anteromedially (Fig. 44)	***N. sudai* Shimizu**
–	Mesoscutum raised along midline (Fig. 29). Crossvein *3rs-m* almost straight (Fig. 31). Disc of propodeum with punctures anteromedially (Fig. 28)	***N. shimizui* Loktionov, Lelej & Xu, sp. n.**
5	Transverse groove on S2 nearly straight (Shimizu et al. 2015: fig. 2E, arrow). T1 with long parallel-sided portion basally (Shimizu et al. 2015: fig. 8C)	***N. iwatai* (Ishikawa)**
–	Transverse groove on S2 subangulate (Shimizu et al. 2015: fig. 6F) or arcuate. T1 without parallel-sided portion basally (Shimizu et al. 2015: fig. 8D)	**6**
6	Mandible short, its apex and two additional teeth rounded, basal tooth vestigial (Shimizu et al. 2015: fig. 8A)	***N. mandibularis* (Ishikawa)**
–	Mandible normal-sized, its apex and two additional teeth pointed, basal tooth distinct (Shimizu et al. 2015: fig. 8B)	**7**
7	Vertex strongly convex between eye tops; hence head in frontal view nearly circular in outline (Shimizu et al. 2015: fig. 1A). Posterior margin of vertex remarkably concave in dorsal view (Shimizu et al. 2015: fig. 1B). Gena strongly developed. F1 length 2.7–2.9× its width. Propodeum gently convex in profile (Shimizu et al. 2015: fig. 1C). S6 not carinate along midline. Fore wing inner fascia along crossvein *cu-a* broad and distinct (Shimizu et al. 2015: fig. 9A)	***N. hayachinensis* (Ishikawa)**
–	Vertex not very strongly convex between eye tops; hence head in frontal view not circular in outline (Shimizu et al. 2015: figs 5A, 6A). Posterior margin of vertex not remarkably concave in dorsal view (Shimizu et al. 2015: figs 5B, 6C). Gena not strongly developed. F1 length 2.1–2.4× its width. Propodeum strongly convex in profile (Shimizu et al. 2015: figs 5C, 6D). S6 carinate along midline. Fore wing inner fascia along crossvein *cu-a* indistinct (Shimizu et al. 2015: figs 9F, 9H)	**8**
8	Ocelli forming right- or obtuse-angled triangle and gena strongly receding posteriorly (Shimizu et al. 2015: fig. 5B)	***N. nagasei* (Ishikawa)**
–	Ocelli usually forming acute-angled triangle and gena roundly receding posteriorly (Shimizu et al. 2015: fig. 6C)	***N. rossicus* (Lelej)**

**Males** (unknown for *N. kurilensis*, *N. mandibularis*, and *N. hayachinensis*)

**Table:** 

1	T1 distinctly petiolate basally (Figs 15, 38); if petiole not distinct (as in *N. sudai*, Fig. 45), then lateral hook on S6 strongly compressed laterally and thin, subtriangular in profile (Fig. 46)	**2**
–	T1 not petiolate basally. Lateral hook on S6 not compressed laterally and not thin, but claw-like, curved and pointed to apex	**4**
2	F3–F11 produced triangularly beneath, forming serrated profile. Lateral hook on S6 large, strongly compressed laterally and thin, subtriangular in profile (Fig. 46). Exposed portion of hypopygium with long erect setae (Fig. 48)	***N. sudai* Shimizu**
–	F3–F11 not produced triangularly beneath, not forming serrated profile. Lateral hook on S6 not compressed laterally nor thin, but claw-like, curved and pointed to apex (Figs 18, 39). Exposed portion of hypopygium without long erect setae (Figs 21, 22, 42, 43)	3
3	Propodeum with fine transverse striae posterolaterally (Fig. 15). Exposed portion of hypopygium narrow (lateral view) (Fig. 22); subbasal portion (ventral view) with round sublateral carina (Fig. 21, arrow)	***N. orientalis* Loktionov, Lelej & Xu, sp. n.**
–	Propodeum without any striae (Fig. 38). Exposed portion of hypopygium wide (lateral view) (Fig. 43); subbasal portion (ventral view) with angulate sublateral carina (Fig. 42, arrow)	***N. shimizui* Loktionov, Lelej & Xu, sp. n.**
4	Ocellar triangle acute- to right-angled. Meso- and metatibial spurs dark brown. Exposed portion of hypopygium compressed laterally with ventral face flattened and polished, broad basally, tapering apically (Shimizu et al. 2015: figs 2F, 8F)	***N. iwatai* (Ishikawa)**
–	Ocellar triangle obtuse-angled; or if right-angled, meso- and metatibial spurs stramineous. Exposed portion of hypopygium completely compressed laterally and very thin, its ventral face linear (Shimizu et al. 2015: figs 5E, 8G–H)	**5**
5	Genitalia with long setae on anterior margin of paramere (Shimizu et al. 2015: fig. 5G)	***N. nagasei* (Ishikawa)**
–	Genitalia with short setae on anterior margin of paramere (Shimizu et al. 2015: fig. 6J)	***N. rossicus* (Lelej)**

## Supplementary Material

XML Treatment for Nipponodipogon

XML Treatment for Nipponodipogon orientalis

XML Treatment for Nipponodipogon shimizui
